# The Effectiveness of the Use of Ultrasound Methodology (Applied to Live Animals) to Assess the Quality of Meat

**DOI:** 10.3390/ani15060872

**Published:** 2025-03-19

**Authors:** Edita Meškinytė, Vigilijus Jukna, Vilma Zigmantaitė, Oksana Ilina, Audrius Kučinskas

**Affiliations:** 1 Animal Production Research and Innovation Center, Agriculture Academy Bioeconomy Research Institute, Vytautas Magnus University, K. Donelaičio g. 58, LT-44248 Kaunas, Lithuania; edita.meskinyte@vdu.lt (E.M.); vilma.zigmantaite@vdu.lt (V.Z.); audrius.kucinskas@vdu.lt (A.K.); 2 Biological Research Center, Lithuanian University of Health Sciences, Tilzes Str. 18/7, LT-47181 Kaunas, Lithuania; 3 Laboratory of Membrane Biophysics, Institute of Cardiology, Lithuanian University of Health Sciences, Sukilėlių Ave. 15, LT-47181 Kaunas, Lithuania

**Keywords:** longissimus dorsi muscle, Black Angus, ultrasound meat quality, beef research, bulls, intramuscular fat

## Abstract

The use of non-invasive technologies is among the most accurate methods for assessing meat quality parameters. Beef tenderness and palatability are strongly influenced by the amount of intramuscular fat, commonly referred to as marbling. Marbling is widely used in breeding programs to select animals with superior meat quality. In this study, we analyzed 236 Angus bulls and 22 Angus heifers from various farms in Lithuania, all aged 450 ± 112 days. Ultrasound examinations were performed using a linear transducer with a silicone standoff to evaluate the loin eye area. Key meat quality indicators, such as fat thickness, loin area and thickness, and intramuscular fat, were found to increase with the weight of bulls, ranging from 365 ± 12.70 kg to 825.57 ± 11.75 kg (*p* < 0.001), and heifers, ranging from 273 ± 20.71 kg to 767 ± 41.01 kg (*p* < 0.001). The highest intramuscular fat values were observed at 7.50 ± 0.75% (*p* < 0.05) to 6.9 ± 1.36% (*p* < 0.1) in bulls weighing 431.33–825.57 kg and 7.98 ± 0.28% (*p* < 0.001) in heifers weighing 603 kg. These findings demonstrate the feasibility of selecting Angus cattle for improved meat quality traits across different weight categories.

## Simple Summary

A novel aspect of this study is the application of ultrasound as a monitoring tool to predict carcass quality in live beef cattle. Researchers and cattle producers utilize ultrasound measurements to evaluate genetic yield and carcass quality in living animals, particularly to identify individuals with superior meat traits. These measurements provide accurate information on carcass performance in living animals without the need for slaughter, enabling further refinement of measures to improve meat quality. Additionally, stress factors during slaughter negatively impact these indicators. By analyzing bulls and heifers of the Black Angus breed from commercial farms in Lithuania, we identified a relationship between weight gain and parameters, such as fat thickness, loin area, loin thickness, and intramuscular fat. It has been established that intramuscular fat was high, starting at weights of 431 kg for bulls and 603 kg for heifers, respectively, suggesting that selection for this trait in Angus is feasible at these ages and weights and is appropriate.

## 1. Introduction

The beef industry is increasingly using live animal carcass ultrasound as a method to control and produce high-quality, consistent products for today’s value-based market [1]. It is considered one of the most cost-effective methods by which livestock producers can improve the genetic traits of carcasses. Ultrasound imaging on live animals allows for determining the meat and fat characteristics, particularly the intramuscular fat percentage and marbling score [1,2].

The most popular breeds of marbled beef are Aberdeen Angus and Black Angus [2]. Black Angus is widely recognized in the market for producing well-marbled, high-quality meat, with exceptional taste and juiciness at a younger age. It is believed that the optimal age for slaughtering this breed is 18 months [1]. According to Travis et al. [3], beef carcass quality is determined by measures such as maturity, marbling, texture, and color.

Ultrasound is the most effective method for assessing meat quality in living animals. Key indicators of good meat quality include fat thickness, the area of longissimus dorsi muscle, hide thickness, the percentage of intramuscular fat (IMF), and the marbling score [4]. Ultrasound is based on the high density of high-frequency sound ultrasonic waves, typically ranging from 2 to 10 MHz, on fat deposits, muscles, and connective and bone tissue. The advantage of such devices is their non-invasive nature, which allows for controlled examination without damaging muscle tissue, while also providing objective measurements and visualizations of muscle and fat beneath the animal’s hide.

Marbling, or intramuscular fat, is a crucial factor in determining the palatability of beef and serves as an important indicator of meat tenderness and juiciness [5]. It can also be utilized in breeding programs to select animals with the potential to pass on superior meat qualities to their progeny [6].

The marbling of beef indicates the ratio of intramuscular fat to the area of the longissimus dorsi muscle [7]. This factor is pivotal in determining the quality of meat, particularly its texture and flavor [8]. The color of beef marbling is influenced by the distribution of fats within the muscle fibers. The marbling score significantly influences the quality and value of beef in various countries, including the United States, Canada, Australia, and Japan. It is believed that the intramuscular fat content in muscles, such as the longissimus dorsi, plays a crucial role in determining and assigning beef quality [9].

The marbling of beef tends to increase as animals age and is influenced by their growth rate and weight [10]. As animals mature, the rate of muscle accretion decreases, while the rate of fat accretion increases quadratically [10,11].

In addition, the flavor and juiciness of beef are affected by the amount of intramuscular fat in the muscle, specifically its ratio to the muscle tissue in the longissimus dorsi. Some scientists have shown that as muscle thickness increases, the juiciness of the meat decreases. Therefore, it is important to consider not only the age but also the breeding and housing conditions of the bulls [12].

The purpose of our research was to analyze meat quality indicators in Angus bulls and heifers using ultrasound in live animals and to demonstrate the impact of weight on meat quality parameters.

## 2. Materials and Methods

The study of the longissimus dorsi muscle was conducted using ultrasound scanning in living animals, a non-invasive technique, with the Esaote MyLab One Vet 8100 112810000 (Oostburg, The Netherlands) portable ultrasound device. The parameter settings included a frequency of 10 MHz and a depth of 5 cm, measured between 2021 and 2023 at the Agriculture Academy Bioeconomy Research, Institute of Animal Production Research and Innovation Center. Our research involved 236 bulls and 22 heifers of the Angus breed from a commercial farm, all at the 450 ± 60 days of age. The animals were grouped by gender and weight, with a 50-kg weight difference between categories. The number of animals in the groups was determined based on those available for the study, and in the heifer group, it was the smallest amount. The number of groups corresponds to the weight categories of the animals. The detailed data of the animals is presented in Table 1.

**Figure 1:**
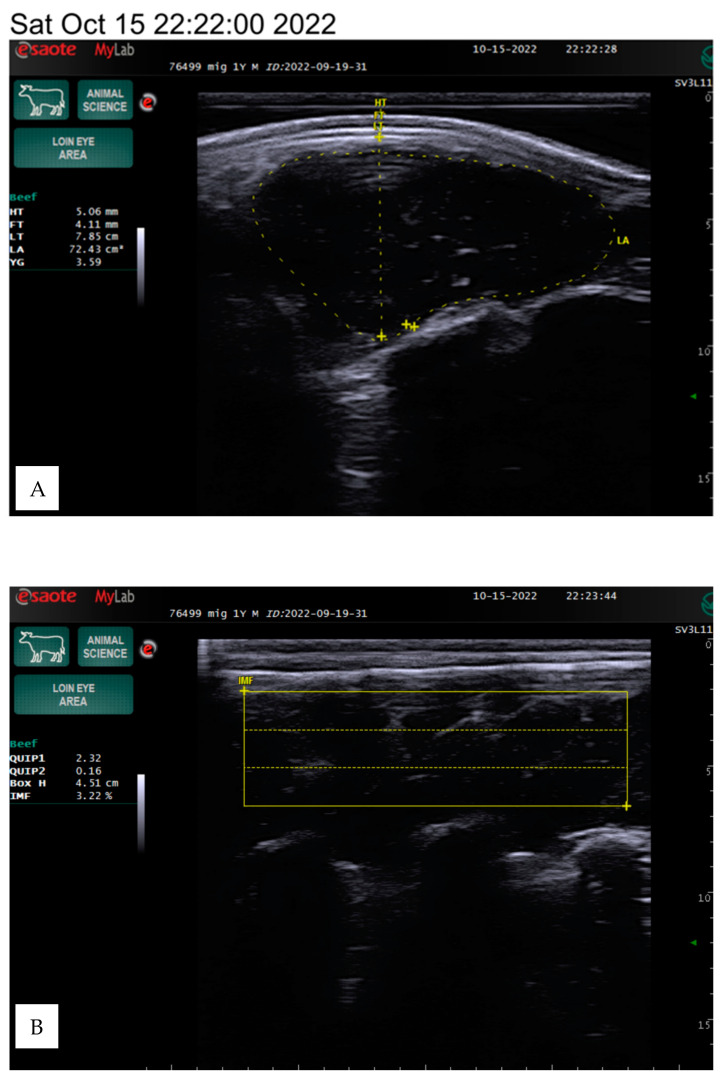

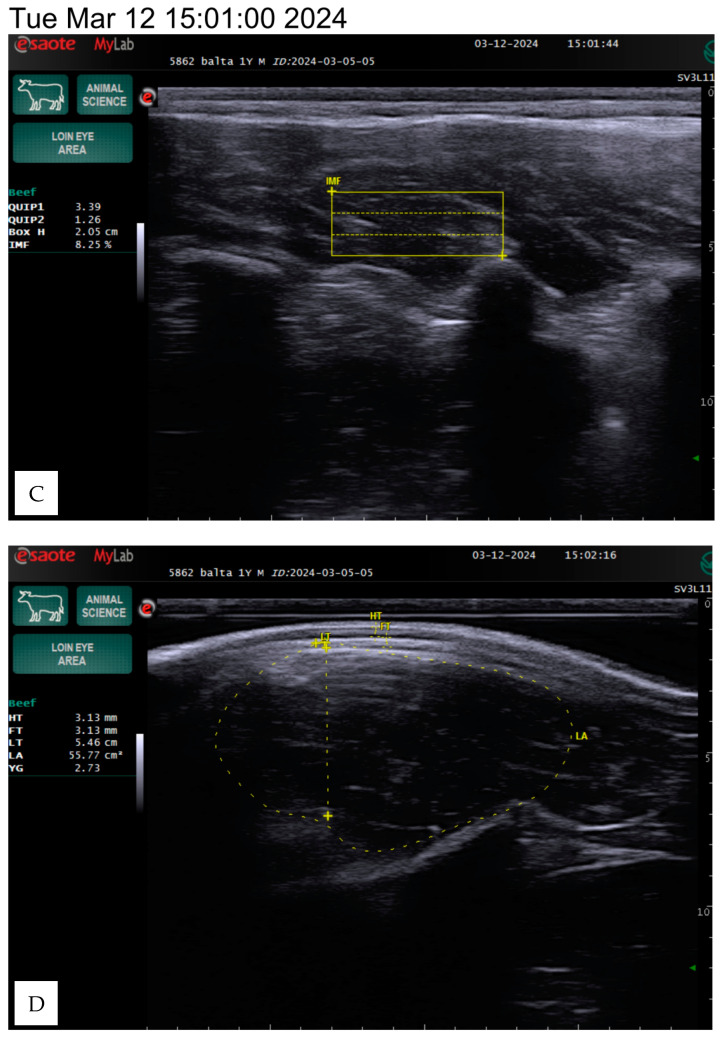
The example ultrasound images of the longissimus dorsi muscle of the Angus bulls, weights of 782 kg, IF 3.59% (**A**,**B**) and 485 kg, IF 8.19% (**B**,**D**). HT—hide thickness, FT—fat thickness (mm), LT—loin thickness (cm), LA—loin area (cm^2^), YG—yield grade, BH—box height (cm), IF—intramuscular fat (%).

The animals were kept under the same conditions and were fed a total mixed ration based on a weight range of 350–600 kg. The ration primarily consisted of 3.5–6 kg of concentrates (barley, maize, oats, peas, and wheat) and 10–20 kg of grass hay, with a protein content of 226–426 g and a dry matter content of 7.21–13.56 kg. The carcass quality indicators were estimated on live animals using an ultrasound linear transducer using a silicone sleeve for the loin eye area with a frequency range of 4.5 to 6.5 MHz and a depth of 8.8 to 13 cm on the 12th and 13th ribs, following the methodology of Silva et al. [13], as presented in Figure 1.

The ultrasound scanning provided a longitudinal view of the 12th and 13th ribs, approximately two-thirds of the distance from the medial to the dorsal end of the longissimus dorsi muscle. After preparing the equipment, the scanner sensor and the surface of the skin were generously moistened with a water-based lubricating gel, and a special silicone tip was applied, and the tip was placed parallel to the rib surface. The examination was completely painless for the animal and did not cause discomfort.

During scanning, the tip was positioned perpendicularly at a 90-degree angle to the surface of the animal’s back muscle and pressed deeply until a smooth, consistent image appeared on the screen. The examination for each animal lasted approximately three minutes. Different structures were visible during the examination: subcutaneous fat, transverse section of the longissimus dorsi muscle, and fat layer near the rib cage. After the examination, stored images were analyzed, and adipose tissue measurements were performed using the scanner. The muscle area and fat layer near the ribs were then assessed.

The subcutaneous fat was measured in millimeters between the 12th and 13th ribs. Interosseous fat, an indicator of external fat, was also measured in millimeters by determining the distance on the screen using the measurement function. The longissimus dorsi muscle area was assessed in square centimeters by outlining the cross-sectional image displayed on the screen.

The area of the longissimus dorsi was measured in square centimeters between the 12th and 13th ribs by determining the perimeter of the cross-sectional image displayed on the screen (Figure 1). The longissimus dorsi muscle is a value of the ratio of muscle to lean product of the animal. Backfat thickness is one of the major quantitative traits that affects carcass quality in beef cattle and has been used to predict carcass retail yield components in live animals. The amount of intramuscular fat determines the indicator, known as marbling.

The intramuscular fat (IF) in the longissimus dorsi muscle was determined using a specialized algorithm developed for the ultrasound device. For this measurement, the silicone tip was removed from the sensor, which was then placed along the muscle. The scan aimed to capture a clear visualization of the three ribs, ensuring high-quality images suitable for evaluation. The measurements were conducted in a dark environment using an ultrasound computer.

The program allowed for the placement of a rectangular grid on the image, which was repeated three times using the three best-quality images. The software then performed calculations based on a predefined formula. The percentage of fat-to-muscle ratio provided an objective assessment of marbling in live cattle, effectively reflecting meat quality.

The evaluation parameters included body weight, hide thickness, fat thickness, loin thickness, loin area, yield grade, box height, and intramuscular fat percentage, which we determined using the Measure mode in the ultrasound program. The box height depends on the screen image and the size of the intercostal area, so the data may vary between animals, as shown in Figure 1.

Statistical analysis was performed using one-way analysis of variance (ANOVA) (Stat 1.1 StatSoft). The parameters for mean and standard deviation follow generally accepted standard statistical methods in Excel (MS Office 2010). The animals were divided into groups, according to weight and gender, for statistical analysis purposes. The mean and standard deviation (Mean ± SD) were calculated for the general indicators, after which the reliability of these indicators was assessed. The arithmetic mean (X), standard error of the arithmetic mean (Sx), and statistical reliability degree of the groups (*p*) were calculated using Stat 1.1 (StatSoft). The reliability of the indicators was evaluated based on probability values by using the statistical analysis software Stat 1: * *p* < 0.1, ** *p* < 0.05, *** *p* < 0.01, and **** *p* < 0.001, and was compared with animals with a live weight of 273 kg for heifers and 365 kg for bulls.

## 3. Results

The quality indicators of the meat were determined by analyzing the ultrasound protocol results obtained from both bulls and heifers, and comparing these results with their respective live weight data.

The meat quality parameters of bulls and heifers are presented in Table 2 and Table 3. Quality indicators, such as fat thickness, loin area, loin thickness, and intramuscular fat (IF), increased with the weight of bulls from 365 ± 12.70 kg to 825.57 ± 11.75 kg (*p* < 0.001) and heifers from 273 ± 20.71 kg to 767 ± 41.01 kg (*p* < 0.001).

The intramuscular fat in the *longissimus dorsi* muscle ranged from 3.36 to 7.97%, with different actual live weights and genders of the animals. So, the best indicators of IF were found in the amounts of 7.50± 0.75% (*p* < 0.05)–6.92 ± 1.36% (*p* < 0.1), with a live weight of 431.33–825.57 kg in bulls, and 7.98 ± 0.28% (*p* < 0.001), with a weight of 603 kg, in heifers. The estimated intramuscular fat was high, beginning with weights of 431 kg and 603 kg for bulls and heifers, suggesting that selection for this trait in Angus is feasible.

In this case, the indicators of fat thickness and the loin area were 2.45 ± 0.66 mm and 53.70 ± 2.92 cm^2^, respectively. However, when the weight increased to 825 kg, the indices of the longissimus dorsi muscle and fat increased significantly (*p* < 0.05), while the intramuscular fat index remained at 6.92 ± 1.36 (*p* < 0.1). This demonstrates the relationship between these indicators and weight. These data indicate performance correlations between bulls and heifers in different weight categories.

In this study, we determined that there was a tendency for an increase in the fat thickness average (range of 2.86 to 6.18 (*p* < 0.05), loin thickness (5.54 to 7.98, *p* < 0.1), and loin area (46.86 to 75.6, *p* < 0.1) in bulls with increased weight. At this time, the highest rate was detected in bulls, with a weight of 825.57 kg. Comparing indicators in the group of heifers, we found a similar dependence on live weight from 273 kg to 767 kg (*p* < 0.001).

Ultrasound measurements allow for predicting the further growth of muscle or fat, which are among the main indicators of meat quality [14,15] and lean meat yield. In our research, this finding demonstrated a significant dependence, with values ranging from 2.51 ± 0.44 to 3.97 ± 0.35 in bulls (*p* < 0.01) with weights of 825.57 kg, and from 2.72 ± 0.12 to 4.05 ± 0.30 in heifers (weights of 767 kg), (*p* < 0.01, *p* < 0.001), indicating muscle growth.

The main feature of the ultrasound method is that these indicators can be predicted during the growth stage in a live animal, allowing for dietary adjustments to improve meat quality.

## 4. Discussion

The results of the impact of live weight on the ultrasonic parameters of intramuscular fat correspond with the data from other authors. According to other researchers, it is extremely important to use efficient methods to estimate parameters related to meat quality, such as in the loin area, fat thickness, and intramuscular fat. The loin area allows us to estimate the grade of yield, as this indicator is related to the amount of meat and muscle. The intramuscular fat percentage has a significant impact on juiciness and flavor, as this indicator is a determining factor related to meat quality and tenderness. The relationship between weight and intramuscular fat was observed at weights starting from 431.33 kg in bulls, and as the weights increased to 775.43–825.57 kg, the intramuscular fat percentage was within the range of 6.92 ± 1.36 (*p* < 0.01). This quality index is a determining factor in beef quality and closely correlates with tenderness.

At present, most of the studies have dealt with the estimation of live animals compared with IF after slaughter. According to previous research [12,16], studies in live animals are not only prevalent but also tend to show higher parameter values compared to post-slaughter measurements. When the animal is slaughtered and the blood is drained, capillaries collapse, reducing the scattering effect of ultrasonic waves [17]. Additionally, the slaughter process induces stress in animals, leading to alterations in meat characteristics.

In our study, the bulls and heifers of the Angus breed were grown under single conditions and subjected to ultrasound examination to determine the parameters of meat quality, namely the important indicator of meat marbling. Our findings demonstrate a clear dependency of marbling indicators on the live weight of the animals. The marbling index depends on the weight of the animals, and the results [10] consistently show that, with an increase in live weight, the area of the longissimus dorsi muscle and the marbling index increase. In our studies, the highest marbling score was observed at 7.50 ± 0.75 (*p* < 0.05) in bulls weighing 431.33 kg, and with an increase in weight to 825.57 kg, this score presented to 6.92 ± 1.36 (*p* < 0.1). Fabbri et al. [18] performed on 27 Charolaise heifers’ carcasses, aged 17 months and 362 ± 59 kg, and detected that the average IF content of meat samples was 5.10 ± 1.44%, which correlates with our research, with values of 3.36 ± 0.70% and weights of 335 ± 15.56 kg. The intramuscular fat in heifers increased to 7.98 ± 0.28 (*p* < 0.001), weighing 603 kg, with a simultaneous increase in measures for the loin area and fat thickness. Similar marbling indices (*p* < 0.10) were determined in 46 bulls weighing 644.8 kg [19] within 3.3–4.2%, with a fat depth of 5.1 mm and longissimus muscle area of 92.9 cm. It was observed that the intramuscular fat content increased to a range of 3.87% to 4.86% as weight increased in 216 Angus-crossbred steers, weighing 270 ± 19.3 kg [20]. These studies show that the percentage of intramuscular fat is variable depending on weight levels and gender. The results of research on carcasses in Bali cattle also showed that the performance of body weight and carcass quality differs between bulls and cows, as well as among age variations [21].

Fiore E. [22] detected a correlation between the actual intramuscular fat percentage in meat during ultrasound analysis and the IF at the time of slaughter, ranging from ≤4.24% to ≥5.76%. The authors assessed the impact of the marbling level and fat percentage on the palatability traits of the meat and detected a relationship (*p* < 0.01) to tenderness, juiciness, and flavor. This has demonstrated that the percentage of intramuscular fat is a more objective measure of marbling content [23].

The authors compared the meat quality and marbling properties of Angus, Simmental, Charolais, and Limousin steers (totaling 416) with an average intramuscular fat content of 3.25% in the longissimus dorsi. According to their findings, clear differences in meat quality were observed between the breeds despite similar IF contents [24,25].

Studies have shown that ultrasound measurements of fat thickness, loin area, and intramuscular fat are accurate indicators of their corresponding carcass traits in fed Angus bulls at slaughter [14]. An evaluation of the ultrasound results on fat thickness, loin area, and the percentage of intramuscular fat showed the results (>0.40) for Angus heifers and bulls at 398 days of age. Ultrasound measured that the intramuscular fat in heifers was 4.22% [26].

Nogalski et al. [6] showed that crossbred bulls, with an average body weight of 520 kg and 18 months of age, had intramuscular fat of 2.33% [2]. The results of the research showed the possibility of predicting the intramuscular fat content in beef in vivo through ultrasound measurements and computer image analysis for Holstein and Slovak Simmental bulls [27,28]. The data established a significant effect of treatment and time of food on the marbling scores (*p* < 0.05) at the location of the longissimus dorsi in Angus, with grades of 3.5 ± 0.5 and 4.6 ± 0.3 for the AM-fed and PM-fed groups, respectively (*p* < 0.05) [29].

Aass et al. [30], in the USA and Australia, have recognized that greater live weight is associated with a larger longissimus muscle area, fat thickness, and the scored meat marbling while ultrasound scanning Angus and Hereford bulls on farms. Ukrainian researchers showed that the ‘muscle eye’ area increases in bulls weighing 400–450 kg at 20–22 months of age due to the impact of age and live weight in Ukrainian Black-and-White Dairy and Ukrainian Meat breeds [16].

Ultrasound scanning of the longissimus dorsi muscle in live animals allows for the identification of individuals with superior beef quality traits, thereby enhancing the breeding value of the cattle herd and enabling further adjustments to their diet [31,32].

## 5. Conclusions

In this study, we demonstrated that ultrasound analysis serves as an effective monitoring tool for predicting carcass quality in live beef cattle. The analysis also revealed notable differences in beef quality traits between bulls and heifers. The intramuscular fat index was significant at 7.50 ± 0.75 (*p* < 0.05) in bulls weighing 431.33 ± 16.74 (*p* < 0.05) kg and in heifers at 7.97 ± 0.40 (*p* < 0.001) weighing 603 ± 5.77 kg, showing the feasibility of the selection of these indicators in Angus at different weights. The significant growth of muscle mass was indicated by the reliability of grade yield in both groups of cattle. Relationships were established between the increases in fat thickness, loin area and thickness, intramuscular fat, and animal weight.

## Figures and Tables

**Table 1:** The sample number of Angus bulls and heifers.

	Sex	Groups of Classes	Groups of Animals
Female (Heifers)			22
Weight category, kg	Below 300	1	7
	300–350	2	2
	350–400	3	7
	400–500	4	2
	600–650	5	3
	650–700	6	2
Male (Bulls)			236
Weight category, kg	350–400	1	4
	400–450	2	6
	450–500	3	24
	500–550	4	40
	550–600	5	46
	600–650	6	53
	650–700	7	29
	700–750	8	13
	750–800	9	14
	800–850	10	7

**Table 2:** Meat quality indicators in bulls of the Angus breed.

	Weight, Mean ± SD, kg/Number of Classes									
	1	2	3	4	5	6	7	8	9	10
Indicators, Mean ± SD	365.00 ± 12.70	431.33 ± 16.74 **	471.58 ± 15.06 ****	521.20 ± 13.75 ****	570.19 ± 13.23 ****	620.15 ± 14.68 ****	669.97 ± 15.15 ****	726.23 ± 12.23 ****	775.43 ± 15.42 ****	825.57 ± 11.75 ****
Hide thickness, mm	5.24 ± 0.80	5.35 ± 0.99	5.22 ± 0.64	5.24 ± 0.74	5.25 ± 0.64	5.12 ± 0.69	5.27 ± 0.64	5.05 ± 0.57	5.05 ± 0.57	4.92 ± 0.41
Fat thickness, mm	2.86 ± 0.71	2.45 ± 0.66	3.60 ± 1.04	3.50 ± 0.94	3.91 ±1.20	4.22 ± 1.26	4.42 ± 1.06	5.09 ± 1.23	5.45 ± 0.99 *	6.18 ± 1.47 *
Loin thickness, cm	5.54 ± 0.78	6.25 ± 0.30	6.40 ± 0.64	6.59 ± 0.81	6.61 ± 0.75	6.90 ± 0.66	6.92 ± 0.85	7.44 ± 0.80 *	7.26 ± 0.59 *	7.98 ±0.72 **
Loin area, cm^2^	46.86 ± 8.62	53.70 ± 2.92	6.40 ± 0.64	60.77 ± 12.39	62.01 ± 8.79	64.68 ± 14.58	63.88 ± 12.37	69.97 ± 11.09	68.14 ± 8.05 *	75.61 ± 6.47 **
Yield grade	2.51 ± 0.44	2.45 ± 0.23	2.62 ± 0.34	2.72 ± 0.69	2.98 ± 0.55	3.24 ± 1.05	3.62 ± 0.90	3.58 ± 0.54	3.97 ± 0.36 **	3.97 ± 0.35 **
Box height, cm	2.96 ± 0.29	3.14 ± 0.52	3.33 ± 0.42	3.30 ± 0.47	3.78 ± 0.71	3.43 ± 0.63	3.45 ± 0.83	3.41 ± 0.49	3.37 ± 0.60	3.28 ± 0.49
Intramuscular fat, %	4.30 ± 1.77	7.50 ± 0.75 **	5.71 ± 2.26	5.16 ± 3.09	5.18 ± 3.29	6.05 ± 2.61	5.71 ± 2.49	4.49 ± 2.23	5.67 ± 2.33	6.92 ± 1.36 *

* *p* < 0.1, ** *p* < 0.05, and **** *p* < 0.001—compared to the animals with a live weight of 365 kg.

**Table 3:** Meat quality indicators in heifers of the Angus breed.

	Weight, Mean ± SD, kg/Number of groups					
	1	2	3	4	5	6
Indicators, Mean ± SD	273 ± 20.71	335 ± 15.56	365 ± 14.6	412 ± 11.31	603 ± 5.77 ****	767 ± 41.01 ***
Hide thickness, mm	4.24 ± 0.57	4.69 ± 0.71	5.58 ± 0.70	5.2± 1.10	5.72 ± 0.45	4.97 ± 0.33
Fat thickness, mm	3.38 ± 0.70	2.7 ± 0.14	3.72 ± 0.81	3.39 ± 0.37	4.34 ± 0.39 **	5.68 ± 0.43 **
Loin thickness, cm	4.58 ± 0.61	4.76 ± 0.08	5.12 ± 0.39	6.02 ± 0.22	5.99 ± 1.12	6.66 ± 0.71 *
Loin area, cm^2^	34.52 ± 2.08	36.21 ± 1.41	44.19 ± 14.35	53.63 ± 7.47	51.30 ± 3.66 **	66.42 ± 9.11 ***
Yield grade	2.72 ± 0.12	2.73 ± 0	3.30 ± 0.70	2.49 ± 0.48	3.66 ± 0.13 ****	4.05 ± 0.30 ***
Box height, cm	2.81 ± 0.28	2.47 ± 0.15	2.77 ± 0.13	2.95 ± 0.74	3.84 ± 0.39	3.39 ± 0.25
Intramuscular fat, %	3.36 ± 0.70	5.61 ± 17.23	3.52 ± 9.85	3.39 ± 0.91	7.98 ± 0.28 ****	5.19 ± 0.65 *

* *p* < 0.1, ** *p* < 0.05, *** *p* < 0.01, and **** *p* < 0.001—compared to the animals with a live weight of 273 kg.

## Data Availability

Data are contained within the article.
